# Draft Genome Sequences of Three Northern German Epidemic *Staphylococcus aureus* (ST247) Strains Containing Multiple Copies of IS*256*

**DOI:** 10.1128/genomeA.00936-16

**Published:** 2016-09-08

**Authors:** Franziska Kleinert, René Kallies, Annegret Zweynert, Gabriele Bierbaum

**Affiliations:** a Institute of Medical Microbiology, Immunology and Parasitology, University of Bonn, Bonn, Germany; b Institute of Virology, University of Bonn, Bonn, Germany; c Helmholtz Centre for Environmental Research, UFZ, Leipzig, Germany

## Abstract

We report the draft genome sequences of three multiresistant *Staphylococcus aureus* strains of sequence type 247 (ST247). The methicillin-resistant *S. aureus* (MRSA) SA1450/94 is vancomycin susceptible, while the clinical MRSA isolate *S. aureus* SA137/93A and its spontaneous laboratory mutant SA137/93G are characterized by intermediate vancomycin susceptibility.

## GENOME ANNOUNCEMENT

Here, we report the draft genome sequences of the multiresistant northern German epidemic (ST247) *Staphylococcus aureus* strains SA1450/94, SA137/93A, and SA137/93G (Table 1). The clinical MRSA isolate SA1450/94 serves as PFGE control strain for the northern German epidemic type (National Reference Center for Staphylococci in Wernigerode, Germany) (ST247) and displays a mutator phenotype that is due to an inactivation of *mutS* by an insertion of IS*256* (1). The heterogeneous vancomycin-intermediate resistant *S. aureus* (VISA) SA137/93A (MIC, 8 µg/ml) was isolated from a tracheal secretion of a patient (2, 3) and the homogeneous VISA strain SA137/93G (MIC, 8 to 16 µg/ml) was selected as a spontaneous laboratory mutant of SA137/93A (2–4).

**TABLE 1:** Accession numbers and properties of the genomes, the versions described in this paper are the first versions of all three sequences

Strain	No. of contigs (>200 bp)	Coverage	Draft genome size (Mb)	No. of annotated genes	Accession no.
*S. aureus* SA1450/94	29	2 to 226	2.94	3,053	JWMI00000000
*S. aureus* SA137/93A	36	0 to 176	2.96	3,103	JWMH00000000
*S. aureus* SA137/93G	41	1 to 132	2.84	2,971	JWMG00000000

The aim of this sequencing project was to find the genetic variations in the VISA strains SA137/93A and SA137/93G in comparison to the vancomycin-sensitive MRSA strain SA1450/94, which may contribute to vancomycin resistance development in both VISA strains. In this regard, previous studies have already shown that the highly active insertion sequence IS*256*, present in the genomes of several clinical isolates of enterococci and staphylococci (5, 6), not only influences vancomycin resistance (4, 7, 8) but also increases the general genome variability in these strains (9, 10).

Genomic DNA of all strains was extracted using the MasterPure Gram-positive DNA purification kit (Epicentre Biotechnologies, Madison, WI). Five hundred nanograms of genomic DNA were fragmented (average size: 350 bp) and a genomic library was constructed according to the GS Junior Titanium Series rapid library preparation method manual (Roche Applied Science, Mannheim, Germany). Fragment end repair, adaptor ligation, and emulsion PCR (Kit Lib-L) were done following the standard Roche protocols. Next generation sequencing (NGS) was performed on a Roche 454-GS Junior system (one run per each sample). Then, NGS reads were *de novo* assembled using the GS De Novo Assembler (Roche) and the Geneious-Assembler 6.0.4 (Biomatters Limited, Auckland, New Zealand). Resulting contigs were reference mapped to the genome sequence of nearest relative, strain *S. aureus* COL (GenBank accession number NC_002951.2). Gap closure and the validation of all IS*256* insertion sites (*S. aureus* SA1450/94: 32 insertions, *S. aureus* SA137/93A: 44 insertions, and *S. aureus* SA137/93G: 38 insertions) were performed using PCR-based techniques followed by Sanger sequencing. Finally, the genomes were annotated using the NCBI Prokaryotic Genomes Automatic Annotation Pipeline (http://www.ncbi.nlm.nih.gov/genome/annotation_prok/). The remarkable accumulation of multiple IS*256* copies in the genomes is due to the fact that transposition is mediated by a copy-and-paste mechanism (11) yielding the highest IS*256* copy numbers of all sequenced *S. aureus* strains yet.

### Accession number(s).

The draft genome sequences of the three *S. aureus* strains have been deposited at DDBL/EMBL/GenBank under the accession numbers described in Table 1. 
